# Inland Treatment of the Brine Generated from Reverse Osmosis Advanced Membrane Wastewater Treatment Plant Using Epuvalisation System

**DOI:** 10.3390/ijms140713808

**Published:** 2013-07-03

**Authors:** Mohannad Qurie, Jehad Abbadi, Laura Scrano, Gennaro Mecca, Sabino A. Bufo, Mustafa Khamis, Rafik Karaman

**Affiliations:** 1 Department of Science, University of Basilicata, Via dell’Ateneo Lucano 10, Potenza 85100, Italy; E-Mails: mqurie@ccba.alquds.edu (M.Q.); sabufo@libero.it (S.A.B.); 2 Department of Chemistry and Chemical Technology, Al-Quds University, Jerusalem, Palestine; E-Mail: mukhamis@yahoo.com (M.K.); 3 Department of Biology, College of Science and Technology, Al-Quds University, Abu Dies, P.O. Box 20002, Jerusalem, Palestine; E-Mail: jihadabbadi@yahoo.com; 4 Department of European Cultures (DICEM), University of Basilicata, Via dell’Ateneo Lucano 10, Potenza 85100, Italy; E-Mail: laura.scrano@libero.it; 5 Exo Research Organization, Potenza 85100, Italy; E-Mail: g.mecca@exo-ricerca.it; 6 Department of Chemistry, Biology and Environmental Sciences, American University of Sharjah, Sharjah, UAE; 7 Department of Bioorganic Chemistry, Faculty of Pharmacy, Al-Quds University, Jerusalem, Palestine

**Keywords:** wastewater treatment, *Ocimum Basilicum*, reverse osmosis, brine, epuvalisation system

## Abstract

The reverse osmosis (RO) brine generated from the Al-Quds University wastewater treatment plant was treated using an epuvalisation system. The advanced integrated wastewater treatment plant included an activated sludge unit, two consecutive ultrafiltration (UF) membrane filters (20 kD and 100 kD cutoffs) followed by an activated carbon filter and a reverse osmosis membrane. The epuvalisation system consisted of salt tolerant plants grown in hydroponic channels under continuous water flowing in a closed loop system, and placed in a greenhouse at Al-Quds University. Sweet basil (*Ocimum basilicum*) plants were selected, and underwent two consecutive hydroponic flowing stages using different brine-concentrations: an adaptation stage, in which a 1:1 mixture of brine and fresh water was used; followed by a functioning stage, with 100% brine. A control treatment using fresh water was included as well. The experiment started in April and ended in June (2012). At the end of the experiment, analysis of the effluent brine showed a remarkable decrease of electroconductivity (EC), PO_4_^3−^, chemical oxygen demand (COD) and K^+^ with a reduction of 60%, 74%, 70%, and 60%, respectively, as compared to the influent. The effluent of the control treatment showed 50%, 63%, 46%, and 90% reduction for the same parameters as compared to the influent. Plant growth parameters (plant height, fresh and dry weight) showed no significant difference between fresh water and brine treatments. Obtained results suggest that the epuvalisation system is a promising technique for inland brine treatment with added benefits. The increasing of channel number or closed loop time is estimated for enhancing the treatment process and increasing the nutrient uptake. Nevertheless, the epuvalisation technique is considered to be simple, efficient and low cost for inland RO brine treatment.

## 1. Introduction

The scarcity of freshwater in most countries of arid and semi-arid regions is an escalating problem, particularly as their populations continue to grow with constant enhancement of their living standards. Water claim is also accelerating due to industrial development and increasing demands of irrigated lands [1,2]. One of the alternative solutions for water scarcity is the use of treated wastewater in agriculture, which simultaneously avoids the negative impact of wastewater disposal in the environment. The reuse of treated domestic wastewater in agriculture has recently expanded and forced some governments for its inclusion in their overall water budget [3,4]. Wastewater treatment technology ranges from traditional low cost treatment to advanced technologies [5]. Advanced wastewater treatment technologies are based on combined processes of biological, chemical and mechanical, which include membrane techniques and disinfection. Advanced membrane technologies consist of microfiltration (MF), ultrafiltration (UF), nanofiltration (NF) and reverse osmosis (RO) processes [6]. These technologies are able to remove particles, turbidity, cysts, bacteria, and even viruses. These sophisticated technologies provide treated water of good quality for unrestricted irrigation so that water resources can be increased and the environment protected [7,8]. The reverse osmosis (RO) process is widely applied after an ultrafiltration process to furnish high quality water for indirect potable and direct non-potable use [9]. Together with high quality water, membrane technologies, including RO process, generates simultaneously a concentrated by-product called brine. Brine is considered as a major problem in treatment plants due to its high salinity and possible content of toxic elements. It cannot be discharged without further purification in order to avoid health problems, environmental complications [10], and pollution of ground water by salts and harmful chemicals [11].

There are various options experienced (legally or illegally) for the treatment and disposal of generated brine including deep well injection, evaporation ponds, disposal into surface water bodies, disposal through pipelines to municipal sewer systems, ion exchange procedures, shrimp breading and hydroponic cultivation of salt tolerant plants (halophytic crops) [9–12]. The reuse of RO-brine in hydroponic agriculture has been proved to be successful using saline effluent up to 6 g total dissolved solids (TDS) per liter with different salt tolerant crops [13,14]. Moreover, this brine was a desirable alternative for irrigation of ornamental and landscape plant cropping [15]. The major and important factors controlling brine reuse in agriculture are salt concentration and brine chemical composition, which can affect soil and ground water quality [16]. Contamination due to toxic elements can pose serious hazards for both humans and ecosystems. The presence of toxic elements in wastewater used for irrigation can cause their accumulation in soil and plants, and consequently can affect food quality. The presence of such elements in the edible part of leafy vegetables should be absolutely avoided because of possible health problems for humans and animals [17–19]. Among other factors playing a role in brine reuse are: vegetation tolerance, land requirements, hydraulic loading rates, site selection and runoff control [20,21].

Epuvalisation is a biological wastewater recycling system based on hydroponic cropping techniques, which could also be employed as a further purification process of effluent water in tertiary sectors of treatment plants. This technique utilizes the roots of plants as bio-filters to remove nitrogen, phosphorus and other macronutrients. In addition, toxic elements and salts can be accumulated into the plant tissues from wastewater as well as brine [22]. The system mechanism consists of gravitational effluent flowing through open channels to keep the water well aerated. The channels host the plant roots not only for water absorption purposes but for trickling and biological filter functions as well. The roots play a dominant role in taking up the nutrients, thus decreasing the total dissolved solids, which includes nitrogen and phosphorus. The technique can be operated in a closed or open loop system. The open loop system is less efficient in the removal of nutrients and salinity due to minimal contact time, while the closed loop system is more efficient because of a relatively longer retention time [23]. The design of channel length, width, depth and slope is very crucial for achieving efficient treatment. The recommended channel’s length for an open circuit is 50 m and for a closed circuit is 10–15 m. The recommended channel’s width in both systems is 50 cm and the depth is 9 cm [23]. The selection of the plant is considered an important and critical factor for the achievement of a successful epuvalistion system. Several ornamental plants, vegetables and grasses were found to be suitable for the purpose, and were selected taking into account the adaptation capacity of each plant species to hydroponic growth and their tolerance to salt [23]. The advantages of the epuvalisation technique include low cost, easy use and flexibility. On the other hand, frequency of plant replacement, energy requirements for water recirculation and cost of the greenhouse (under temperate climate) are considered as major disadvantages of this technique [23].

The objective of this study was to investigate the potential of using an epuvalisation system to treat brine generated from an inland RO unit. The system utilizes Basilicum (*Ocimum basilicum* L.) as the salt tolerant plant. Water quality parameters as well as plant growth factors were monitored and the objective of reaching zero liquid discharge (ZLD) was achieved. The ZLD consists in the elimination of any liquid discharge from the wastewater treatment plant (WWTP) by its purification and recycling [24]. This process can be advantageous from both an economical and ecological point of view.

The novelty in our study is the treatment of RO-brine, characterized by high EC (mS cm^−1^), using Basilicum as a salt tolerant plant. We aimed at prior purification of RO brine water to permit its reuse. To our knowledge, no papers were previously published on the purification of RO brine using epuvalisation systems. The aim of existing works was normally the direct reuse of brine for plant irrigation, avoiding its purification and eventual recycling in the WWTP. In other cases the epuvalisation system was used for the direct treatment of wastewater with lower EC compared to RO-brine.

## 2. Results and Discussion

### 2.1. Results

#### 2.1.1. Characteristics of RO Brine

Chemical, physical and biological characteristics of the RO brine used in the epuvalisation system are presented in Table 1. Brine was rich in all major ions and its EC value was almost doubled in comparison to wastewater (Table 2). This is not surprising, since this water is a concentrate of all rejected ions from the RO unit. On the other hand, brine does not contain pathogens, which are eliminated in the ultra-filtration sector. In fact, water input to the RO unit came from the permeate of the ultra-filtration sector furnished with 20 kD cutoff filters (spiral-wound unit), which has the capability to remove bacteria and viruses.

#### 2.1.2. Brine Quality during Epuvalisation Treatment

The chemical and physico-chemical characteristics of water flowing out from each sector of the treatment plant are presented in Table 2. Careful examination of Tables 1 and 2 reveals that the RO brine is two-fold more concentrated than the effluent brine from the activated sludge stage. Our aim in this work is to reduce the concentration of RO brine by the epuvalisation technology to a level that is equal to or less than the effluent from activated sludge. This is in order to enable the water to be recycled within the system for further reuse, thereby achieving ZLD and minimizing its negative effects on the environment.

The influent and effluent quality of brine was monitored during the epuvalisation experiment. Water samples were collected and analyzed after 14 days (50% brine) and 28 days (100% brine) from plantation. Results are presented in Tables 3 and 4. Examination of both tables reveals that in 50% brine treatment (Table 3) the EC, TDS and chemical oxygen demand (COD) values were reduced to half of their initial values, while biological oxygen demand (BOD) was reduced to 66%. The reduction of ammonium-N was higher than that of nitrate-N. Sodium ion displayed the highest percentage of plants’ uptake (48%) followed by K^+^ (25%) and Ca^2+^ (14%). Chloride and phosphates were reduced up to 66% and 22% of the initial concentration, respectively. In the control treatment, the EC and TDS values were reduced by 63 and 57% of their concentrations in fresh water, respectively. COD and BOD were reduced to 50% of their concentrations. As in the brine treatment, ammonium-N and nitrate-N were reduced, but the removal of the former was significantly higher. The plants were found to remove K^+^ in a higher extent than Na^+^ (opposite to brine treatment) and Mg^2+^. Chloride was reduced to about 30%. Although PO_4_^3−^ concentration was low, its removal was quite high (77%).

Using 100% brine (Table 4), the EC and TDS were reduced to 42% and 52%, respectively. COD was reduced to a greater extent than the BOD values. While almost 98% of ammonium-N was removed, only 10% of nitrate-N was utilized by plants. Most of the Na^+^ was removed from brine (83%), while only 60% of K^+^. The concentrations of Mg^2+^ and Ca^2+^ were reduced by 26% and 11%, respectively, while 32% of chloride was removed and most of PO_4_^3−^ was taken up by the plants. In the control treatment using nutrient solution in fresh water, EC and TDS values were halved. BOD was reduced more than COD. Most of the ammonium-N and nitrate-N was removed. Potassium was reduced 90% of initial concentration, while the Na^+^ was reduced 40%. Chloride and phosphates were reduced 27% and 64%, respectively.

#### 2.1.3. Real Time Analysis of EC

**Figure 1:**
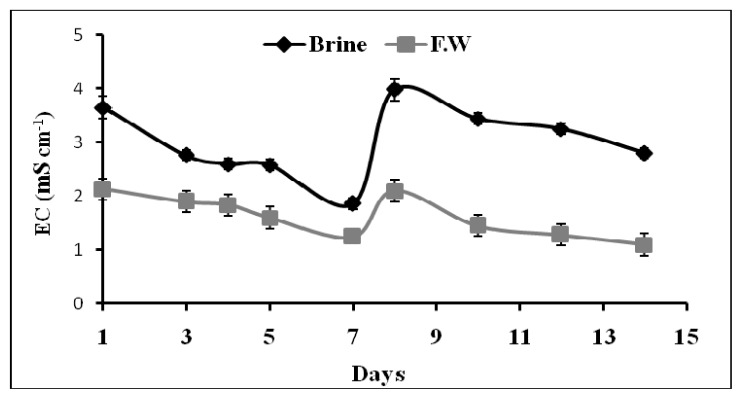
Variation of EC *vs.* epuvalisation time for two cycles of seven days each using two reservoir recharges with the 1:1 mixture brine:fresh water. Fresh water results were included as a control. Both treatments contained the same quantity of macro and micro nutrients. Mean values ± standard deviations (SD) of three replicates.

**Figure 2:**
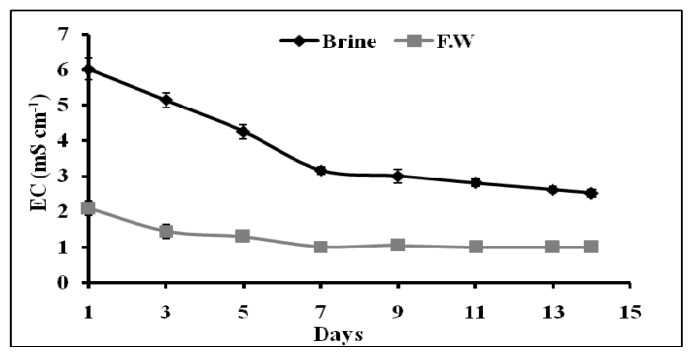
Variation of EC *vs.* epuvalisation time using 100% brine, compared to treatment with fresh water as control. Both treatments contained the same quantity of macro and micronutrients. Mean values ± standard deviations (SD) of three replicates.

Figure 1 illustrates the variation of EC (mS cm^−1^) *vs.* the time during the adaptation period in which the brine used in the closed loops was mixed at a 50% rate with fresh water. On the 1st and 7th day from the beginning of the hydroponic cycle two doses of the same quantity of fertilizers were added both to the tank containing the mixture brine/fresh water and the tank containing only fresh water. Results for both cases are included in Figure 2 for comparison. Data shows that after the addition of fertilizers, there is a gradual decreasing of EC values during the monitoring period. This reduction can be attributed to the plant uptake. When the plants were irrigated with the 100% brine water (Figure 2) the EC was found to decrease with time as well. Similar results were observed for the control. The overall decrease at the end of the experiments was more than 60% in both trials using either 50% brine (mixed to 50% fresh water) or 100% brine as irrigation water. This finding indicates that water effluent from epuvalisation system can be considered of the same quality as the water effluent from the activated sludge unit, as reported in Table 2. This water can be recycled directly into UF and RO sectors of the plant for further purification without the need of extra pressure, thus achieving ZLD strategy.

#### 2.1.4. Plant Growth Parameters

**Figure 3:**
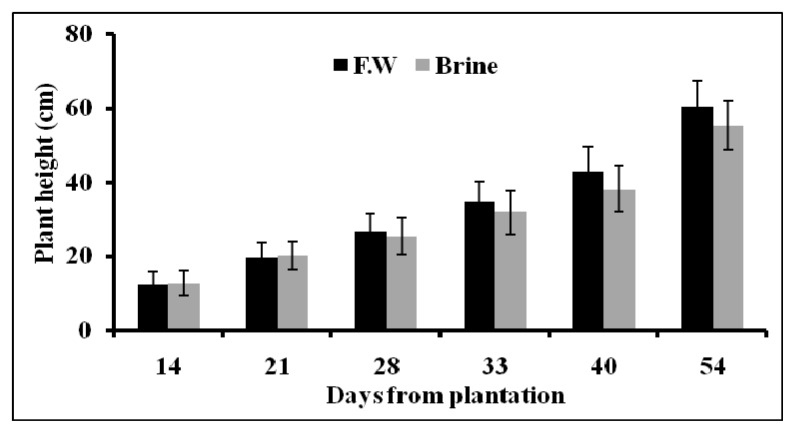
Variation of Basilicum height after 14 days of epuvalisation experiment using fresh water, 14 days using the 1:1 mixture brine:fresh water, and other 26 days using 100% brine, compared to treatment with fresh water as control. Mean values ± standard deviations (SD) of three replicates.

**Figure 4:**
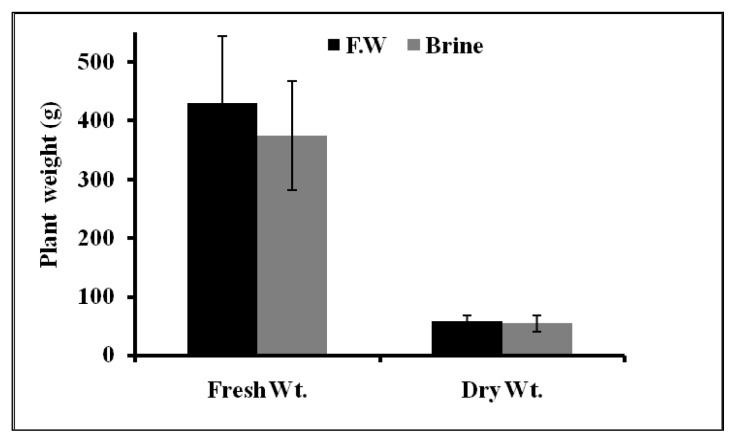
Basilicum fresh and dry weight after 14 days of epuvalisation experiment using fresh water, 14 days using the 1:1 mixture brine:fresh water, and other 26 days using 100% brine, compared to treatment with fresh water as control. Mean values ± standard deviations (SD) of three replicates.

The plants grew very well in the hydroponic system. Plant growth parameters (plant height, fresh weight and dry weight) of Basilicum cultivated in brine and fresh water hydroponic system is summarized in Figures 3 and 4. The plant height was found to increase normally with time. There was no significant difference between plant height in the case of brine and fresh water treatments. This finding is not surprising since Basilicum plants can tolerate saline water application up to 80% salt without any significant reduction of plant height [25]. No significant change of fresh and dry weight of Basilicum plants was observed between both treatments.

#### 2.1.5. Chemical Composition of Plant Tissues

**Figure 5:**
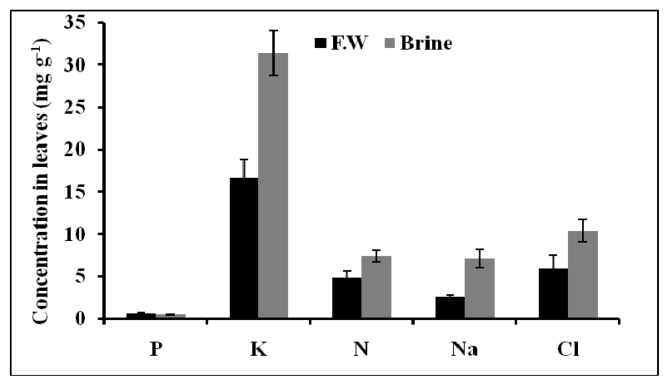
The total nutrients content of Basilicum leaves after 14 days of epuvalisation experiment using fresh water, 14 days using the 1:1 mixture brine:fresh water, and other 26 days using 100% brine, compared to treatment with fresh water as control. Mean values ± standard deviations (SD) of three replicates.

**Figure 6:**
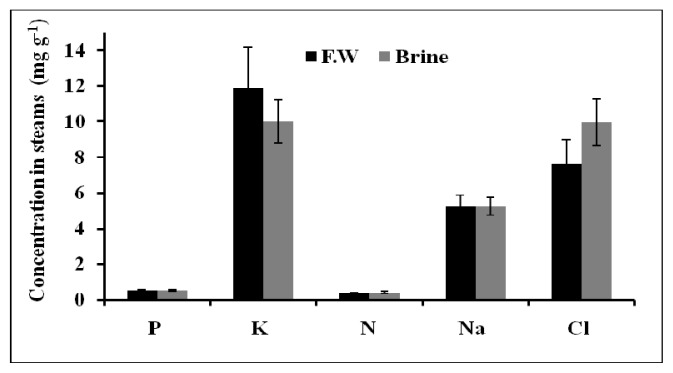
The total nutrients content of Basilicum stems after 14 days of epuvalisation experiment using fresh water, 14 days using the 1:1 mixture brine:fresh water, and other 26 days using 100% brine, compared to treatment with fresh water as control. Mean values ± standard deviations (SD) of three replicates.

Basilicum plants grown in brine accumulated a higher Na^+^, K^+^, and Cl^−^ amount in their leaves compared to plants irrigated by using only fresh water (Figure 5). But both brine and fresh water treated plants accumulated the same amount of N and P. Basilicum grown in brine water was found to accumulate much more Na^+^ in the stems than plants grown in fresh water (Figure 6).

**Figure 7:**
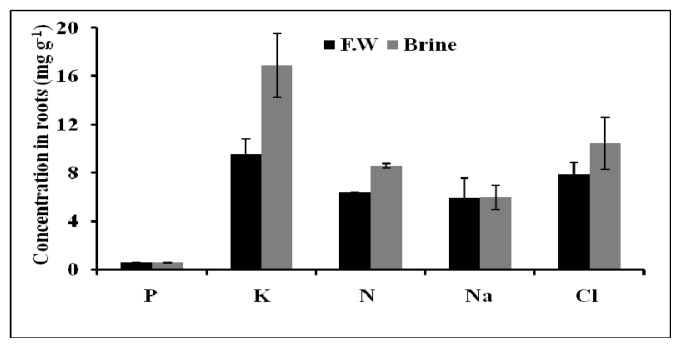
The total nutrients content of Basilicum roots after 14 days of epuvalisation experiment using fresh water, 14 days using the 1:1 mixture brine:fresh water, and other 26 days using 100% brine, compared to treatment with fresh water as control. Mean values ± standard deviations (SD) of three replicates.

On the other hand, the same amounts of K^+^, Cl^−^, N, and P were found in the plant stems in both cases. Similarly, roots of Basilicum grown in both brine and fresh water accumulated independently the same amount of the following elements: Na^+^, K^+^, Cl^−^, N, and P (Figure 7).

The macronutrients N, P and K^+^ in leaves, stems and roots of Basilicum grown in brine and fresh water did not show any significant difference between both waters. Potassium was accumulated in higher amounts compared to the other macronutrients. The high content of K^+^ was recorded during vegetative stage for all plants. Sodium content in roots was found not to differ in plants treated with both waters. No particular accumulation of chloride was observed in the roots, stems and leaves of the Basilicum plants.

### 2.2. Discussion

Techniques involving the recirculation of the wastewater after biological treatment and/or filtration promote hydroponic systems as methods with a high potential for the treatment and reuse of wastewater [26]. Substantial research has been done on the use of plants in wastewater treatment using different techniques such as algae culture, floating emerged or submerged plant culture, raft or suspended culture, nutrient film technique (NFT), aeroponics and static culture (combination of inert medium and water culture) which represent different water culture designs [27]. Although some of these systems are effective, the production of large quantities of aquatic plants with low economic value poses a big challenge [28]. Thus, crops can be cultured hydroponically in recirculating systems to produce a valuable by-product, while improving the water quality to a highly desired level. High-value vegetable crops, such as tomatoes, lettuce, cucumbers and sweet basil, have been cultured in a variety of hydroponic media [29].

Results of this study revealed the plants under study were found to grow very well in the hydroponic system. The concentration of dissolved solids in brine declined during the plants growth period. This is might be attributed to the ability of plant roots, as they develop, to act as filters thus reducing the dissolved solids values in the effluent. The root system was also capable of absorbing dissolved solids as plant nutrients. The reduction of TDS from brine water as an effect of plant uptake was 50%.

In this study, the percentage of COD reduction by plant activity in both diluted and not diluted brine was 47% and 67%, respectively. The large decrease in COD could be related to their fully developed plant roots, which effectively act as filter for suspended solids and absorb dissolved nutrients, thus leading to this significant reduction. Jiang and Xinyuan [30] documented that 44% reduction of COD was achieved in zoo wastewater using floating (water hyacinth and mosquito fern), submerged (curly pondweed, eelgrass and parrot feather), floating leaf (hindulotus) and emerged (swamp morning-glory and alternanthera alligator) plants.

The nitrate-N concentration in the final effluent was reduced by plant absorption. The rate of this reduction was found to increase as plants grow and develop. The plants had a high content of nitrogen in the first growth stages while the nitrogen content decreased in the seed-setting stage [31]. Although the reduction of NO_3_^−^ nitrogen was only 26% and 10% of the influent concentration containing either 50% or 100% brine, respectively, the total NO_3_^−^ removal was 107 and 96 g in the respective treatments. On the other hand, the total reduction of ammonium-N was between 96% and 98%. This percentage corresponds to a total removal of 140 and 336 g in 50% and 100% brine, respectively. The high removal of ammonium-N during the epuvalisation system could be attributed to both direct plant uptake by the plants and/or nitrification. Gloger *et al.* [31] reported that lettuce was responsible for the removal of 9% of nitrogen applied as feed, while Jiang and Xinyuan [30] documented a 73% removal of total nitrogen of zoo wastewater (110 kg total nitrogen/year) using different plant types. Furthermore, Naegel [32] reported a nitrate level reduction in aquaculture wastewater of about 78% (from more than 450 to about 100 mg/L) in 8 weeks when using tomatoes and about 89% (from more than 450 to about 50 mg/L) in 4 weeks when using lettuce. On the other hand, Mantet *et al*. [33] reported 58% nitrogen removal using Salix viminalis grown in gravel hydroponic system to treat primary settled sewage wastewater.

Phosphorous is considered to be a major growth-limiting nutrient in aquatic systems [34]. Wastewater application was therefore a beneficial source of phosphorous required by plants. Under normal conditions, phosphorous occurs either as orthophosphate (HPO_4_^2−^ or H_2_PO_4_^−^) ions or organic compounds dissolved in water. Phosphorous is converted from inorganic to organic and vice versa, by microorganism activity. Biologically available phosphorous in aquatic system includes soluble reactive and unreactive phosphorous. The latter is available as a result of enzymatic hydrolysis [35]. Our results indicate that about 75% of the applied P was removed in both 50% and 100% brine treatments. The total amount of P removed by plants as PO_4_^3−^ was low (0.83 g and 1.33 g in both 50% and 100% brine treatment, respectively). Nevertheless, the plants did not show any P deficiency symptom [36]. Jiang and Xinyuan [30], achieved 62% removal of total phosphorous from zoo wastewater using different plant types. Other investigators [37] reported a removal efficiency of 99% and 97% of total and soluble phosphorous, respectively, in saline aquaculture wastewater using salt tolerant plants grown in a sand biofilter. Further, Gloger *et al.* [38] reported a decrease of PO_4_^3−^ concentration in fish wastewater circulated in a hydroponic lettuce tank of 4.9 mg/L. On the other hand, Mant *et al.* documented that 90.6% phosphorous removal was achieved using *S. viminalis* grown in a gravel hydroponic system to treat primary settled sewage wastewater [33].

Potassium concentration in each compartment decreased with time during the growth period. The concentrations listed in Tables 2 and 3 indicate a potassium removal of about 25 and 60 in the 50% and 100% brine treatments, respectively. The total amount of K^+^ removed by plants from 50% and 100% brine treatments was 15 g and 33 g, respectively. Our results are in agreement with those obtained by Dontje and Clanton [39], which reported 25%–71% potassium removal in circulating aquacultural systems using cattails, reed canary grass, and tomatoes grown in sand beds. Furthermore, Mant *et al.* reported 25% potassium removal using *S. viminalis* grown in a gravel hydroponic system to treat primary settled sewage wastewater [33]. The removal efficiency of potassium achieved in this and other studies could be attributed to plant uptake.

The brine wastewater used in our system, was rich in potassium, which resulted in potassium over fertilization for all plants (Table 4). It was indicated that when potassium supply is abundant, “luxury consumption” of potassium often occurs, which may have a possible interference with physiological availability and uptake of magnesium and/or calcium [35]. It is worthy to note, that it is well known that high K^+^ level in soil and fertilizer is considered as good character for high productivity [40,41].

## 3. Experimental Section

### 3.1. Wastewater Treatment System and Site

The wastewater treatment plant at Al-Quds University was selected to perform this study. The details of the treatment plant were described in previous investigations [42,43]. Briefly, it consists in a sequence of activated sludge unit, hollow fiber ultra-filtration filters (HF-UF), spiral wound ultra-filtration filters (SW-UF), granular activated carbon (GAC) and RO filters. The RO system consists of a 1 × 4 inch pressure vessel constructed from composite material having a pressure resistance up to 400 psi. The vessel holds two 4 inch separation membranes composed of thin polyamide film with pH range 1–11 (model BW30-4040 by DOW Filmtec, Edina, MN, USA). A membrane-antiscaler (product NCS-106-FG) solution (phosphoric acid disodium salt) is continuously dosed to the RO feed at a concentration of 4 mg L^−1^ in order to prevent deposition of divalent ions in the RO membrane. The system is designed to remove major ions and heavy metals. The intended RO permeate capacity of the system is 0.45–0.50 m^3^ h^−1^. The quantity of RO brine generated from the plant is estimated as 25% of the total flow. It is collected in special storage tank for further treatment and reuse.

### 3.2. Epuvalisation System

**Figure 8:**
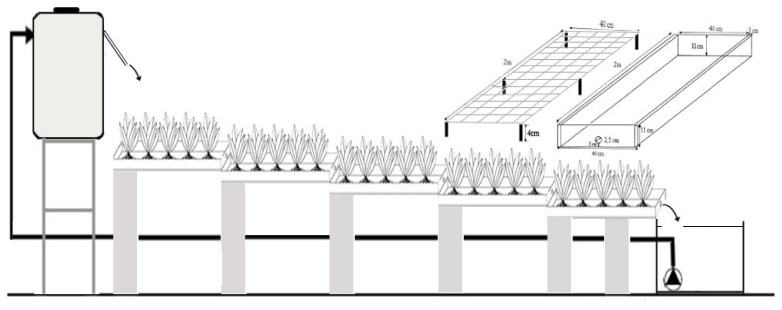
Graphical design of the epuvalisation system.

The experiment was conducted in a greenhouse from April to June 2012 at Al-Quds University under semi-controlled conditions (day and night temperatures were maintained at 25 °C and 18 °C, respectively, and relative humidity of 50%–60%. Epuvalisation system (Figure 8) is composed of two equivalent sectors; in the first, brine was used as hydroponic water, and the second was adopted as a control device employing only fresh water. Each system (brine treatment or fresh water treatment) consists of two 0.5 m^3^ storage tanks (one for influent and the other for effluent water) and 5 cropping channels. For both treatments (brine and fresh water), the influent tank is placed one meter higher than the epuvalisation tracks to let water flow by gravity through five channels. Each channel (made of galvanized metal) is 2-meter long, 40-cm wide and 11-cm deep. The five channels are placed consecutively with 10-cm height difference between each channel. The slope of each channel is about (1%–1.5%). The effluent storage tank is located under the channel placed in the bottom. The effluent was continuously pumped to the influent tank to close the cycle. To enhance the dissolution of oxygen, the channels were continuously aerated by means of a pump and thin aeration plastic pipes.

### 3.3. Plant Selection

Basilicum (*Ocimum Basilicum* L.) is an annual herb plant from the Lamiaceae family; it is considered a medicinal plant and used often as a spice. The plant contains an essential oil, which can be used in manufacturing perfumes and flavors for food and beverages [44,45]. Basilicum has restricted requirements of water and minerals [31]. Basilicum was used in epuvalisation systems by other researchers along with different plant species such as: Metha, Mint, Peppermint, Alfalfa, Sudax, Salvia, Gerbera, Turf Grass, Celery, Cyperus, Water Cress and Iris [15,19].

### 3.4. Plantation and Growth

Young plants of Basilicum were grown in aerated brine and fresh water supplied with additional nutrients. Nutrients added in the influent tanks of brine and fresh water treatments were 5.0, 4.0, 1.0, 0.8, 0.7, 0.5 mM total nitrogen, total potassium, calcium, phosphorus, magnesium, and iron, respectively, using the following chemicals: K_2_SO_4_, KCl, KNO_3_, Ca(NO_3_)_2_·4H_2_O, NH_4_NO_3_, KH_2_PO_4_, MgSO_4_·7H_2_O, Fe–Na EDTA. Micronutrients were added in adequate amounts (μM): 2.97 MnCl_2_·4H_2_O, 1.24 ZnCl_2_, 0.66 CuCl_2_·2H_2_O, 24.75 H_3_BO_3_, 0.083 (NH_4_)_6_ Mo_7_O_24_·4H_2_O, and 0.0413 NiCl_2_[40]. The plant cropping in the brine irrigated channels was performed following a time based program: fresh water, during the first 2 weeks; 50% brine, other 2 weeks for plant adaptation; 100% brine to conclusion of experiment. In the second sector only fresh water added with fertilizers (macro- and micro-nutrients) was used as hydroponic water during the same experimental time of the first sector.

The nutrients were added once at the beginning of the experiment in the inlet container. The flow rate in the system was maintained daily at 0.5 m^3^ influent per 24 h. Basilicum plants were put at 25 cm distance each other to permit roots to develop in a sufficient volume (seven plants in each channel), so 35 replicates were performed in each cropping system.

### 3.5. Water Analysis

Water samples were taken weekly from influent and effluent tanks up to the end of cropping period. Electrical conductivity EC, pH, total dissolved solids (TDS), chemical oxygen demand COD, biological oxygen demand BOD, and most important ions (Cl^−^, NO_3_^−^, NH_4_^+^, Na^+^, K^+^, Ca^+2^, Mg^+2^, PO_4_^−3^) were analyzed according to standard methods [46].

### 3.6. Harvesting and Analytical Procedures

Plant growth parameters were monitored at the end of the growing period (plant height, fresh weight, and dry weight). Plants were harvested in flowering growth stage 57 days after planting (DAP). Each plant was harvested individually. Plants were separated into leaves, stems, and roots. All plant parts were dried at 70 °C in a drying oven until constant weight, grinded to pass a 1.5 mm sieve. After thorough mixing, a sub-sample of 5 g was milled to a fine powder. Plant materials were prepared to be analyzed for P, K and Na using dry ashing method, in which 50 mg of dried sample were ashed in a crucible at 450 °C in a muffle furnace overnight, then 1 mL of 0.35 M HNO_3_ solution was added, swirled and left for at least 10 min. Pure water (18.2 MΩ cm^−1^) was added (9 mL) and then the sample was filtered through ashless filter paper (Whatman International Ltd., England) into polypropylene tubes [47]. Total P was measured using colorimetric method (Ammonium-Vanadate-Molybdate) according to Gericke and Kurmies [48]. Total N was determined using Kjeldahl method [49].

### 3.7. Statistics and Yield Component Analysis

Results were expressed as means and standard deviations for three replicates. All statistical analyses were carried out using Statistical Analysis System (SAS) (SAS Institute Inc., Cary, NC, USA, Release 8.02, 2001). Comparison among data was carried out using the GLM procedure. The Bonferroni procedure was employed with multiple-tests in order to maintain an experiment wise of 5%.

## 4. Conclusions

Soil salinity, surface and ground water contamination are the major problems of brine water generated from a RO plant. The management of the brine water problem will help in minimizing health and environment risks. The reuse of brine in irrigation of high salt tolerant plants is considered among the most suitable disposal methods to solve the brine problem. In this work, we demonstrated that the newly developed epuvalisation technique has not only high potential in reducing the salt content of brine to a safe level, but also to produce plants of high economic value without any loss of yield. Basilicum was selected for this purpose and our results showed that it can be grown in brine generated from the RO system with high tolerance and good yield. Results show a significant decrease in the electrical conductivity in brine during the growing season. Plant growth parameters of Basilicum irrigated with brine and fresh water have shown insignificant difference between them. This work demonstrates that the epuvalisation system is simple, flexible, easily managed and low cost. It has a high potential for inland brine treatment if a proper salt tolerant plant, such as Basilicum, is used.

## Figures and Tables

**Table 1:** Chemical, physical and biological parameters of brine water generated from the RO unit.

Parameter	Mean value ± SD (different units) a	Ions	Mean value ± SD (mg L^−1^)
pH	7.70 ± 0.30	Cl^−^	2,560 ± 80
EC	4.50 ± 0.50	NO_3_^−^	95.0 ± 5.0
TDS	2,250 ± 500	PO_4_^3−^	2.30 ± 0.70
COD	330 ± 55	NH_4_^+^	720 ± 20
BOD	120 ± 20	Na^+^	330 ± 25
FC	0	K^+^	81.0 ± 10
TC	0	Ca^2+^	154 ± 15
		Mg^2+^	59.0 ± 13

a EC, electrical conductivity (mS cm^−1^); TDS, total dissolved solids (mg L^−1^); COD, chemical oxygen demand (mg L^−1^); BOD, biological oxygen demand (mg L^−1^); FC, fecal coliforms (cfu mL^−1^); TC, total coliforms (cfu mL^−1^); SD, standard deviation of three replicates.

**Table 2:** Physical, chemical and biological characteristics of influent and effluent wastewater by different treatment units, which include activated sludge (AS), ultrafiltration-hallow fiber (UF-HF), ultrafiltration-spiral wound (UF-SW) and reverse osmosis (RO). Mean values ± standard deviations (SD) of three replicates.

Parameter	Influent a	AS b	UF-HF	UF-SW	RO
pH	7.50 ± 0.30	7.12 ± 0.47	7.50 ± 0.07	7.45 ± 0.08	6.20 ± 0.15
EC (mS cm^−1^)	1.99 ± 0.23	1.90 ± 0.22	1.53 ± 0.01	1.53 ± 0.01	0.03 ± 0.01
TDS (mg L^−1^)	966 ± 105	912 ± 92	760 ± 10	750 ± 16	30 ± 12
COD (mg L^−1^)	380 ± 150	182 ± 96	90 ± 20	46 ± 13	20 ± 11
BOD (mg L^−1^)	242 ± 138	107 ± 49	56 ± 25	41 ± 14	10 ± 5
Cl^−^ (mg L^−1^)	268 ± 62	192 ± 100	246 ± 20	246 ± 10	13.50 ± 1.05
NO_3_^−^ (mg L^−1^)	15.50 ± 10	12.40 ± 10	10.02 ± 5.02	13.03 ± 3.06	2.50 ± 0.10
PO_4_^3−^(mg L^−1^)	25.69 ± 3.15	14.70 ± 2.2	10.16 ± 1.50	3.14 ± 0.50	0.23 ± 0.05
NH_4_^+^ (mg L^−1^)	91 ± 64	89 ± 56	85 ± 11	23.30 ± 3.11	5.20 ± 0.21
Na^+^ (mg L^−1^)	128 ± 45	86 ± 50	112 ± 14	114 ± 10	7.30 ± 2.12
K^+^ (mg L^−1^)	41 ± 30	36 ± 13	38.8 ± 10	19.70 ± 5.05	1.38 ± 1.03
Ca^2+^ (mg L^−1^)	65 ± 21	60 ± 15	65 ± 12	65.70 ± 13	1.75 ± 0.51
Mg^2+^ (mg L^−1^)	30 ± 10	24 ± 20	24 ± 11	27.30 ± 5.01	0.68 ± 0.11
TC (cfu/mL)	(6 × 10^5^) ± 10^2^	(2 × 10^4^) ± 10^1^	120 ± 50	0	0
FC (cfu/mL)	(5 × 10^3^) ± 10^2^	(2 × 10^2^) ± 10^1^	40 ± 20	0	0

a Initial composition of wastewater to be treated;

b The effluent from AS is the influent to UF-HF, and so on.

**Table 3:** Physical and chemical quality of hydroponic recycled water after 14 days of epuvalisation treatment with fresh water and 14 days with the 1:1 mixture brine:fresh water, compared to treatment with only fresh water during the same period. Mean values ± standard deviations (SD) of three replicates.

	Brine: Fresh Water 1:1 (*v:v*) a			Fresh Water a		
		
Parameter	Influent	Effluent	% removal	Influent	Effluent	% removal
pH	7.30 ± 0.1	7.8 ± 0.2		7.40 ± 0.1	7.5 ± 0.10	
EC (mS cm^−1^)	3.82 ± 0.2	1.91 ± 0.2	50.0	1.74 ± 0.1	1.10 ± 0.1	37.0
TDS (mg L^−1^)	1910 ± 120	930 ± 113	51.0	905 ± 63.0	520 ± 40.0	43.0
COD (mg L^−1^)	141 ± 15.0	75 ± 10.0	47.0	73.0 ± 10.0	38 ± 10.0	48.0
BOD (mg L^−1^)	61 ± 10.0	20 ± 10.0	67.0	40.0 ± 10.0	19 ± 10.0	53.0
Cl^−^ (mg L^−1^)	1631 ± 500	1081 ± 200	34.0	420 ± 80.0	285 ± 60.0	32.0
NO_3_^−^ (mg L^−1^)	838 ± 231	624 ± 19.0	26.0	645 ± 10.0	332 ± 74.0	49.0
PO_4_^3−^ (mg L^−1^)	2.15 ± 0.1	0.50 ± 0.1	77.0	2.15 ± 0.1	0.50 ±0.10	77.0
NH_4_^+^ (mg L^−1^)	292 ± 80.0	12 ± 4.0	96.0	44.0 ± 4.00	10 ± 4.0	77.0
Na^+^ (mg L^−1^)	192 ± 7.0	100 ± 49.0	48.0	54.0 ± 2.0	43 ± 2.0	20.0
K^+^ (mg L^−1^)	123 ± 19.0	93 ± 6.0	25.0	110 ± 2.0	66 ± 26.0	40.0
Ca^2+^ (mg L^−1^)	117 ± 2.0	101 ± 1.0	14.0	79.0 ± 2.0	80 ± 1.0	0.0
Mg^2+^ (mg L^−1^)	55 ± 1.00	46 ± 4.0	17.0	37.0 ± 1.0	32 ± 1.0	14.0

a Brine flowing from RO unit. Macro- and micro-nutrients were added as described in “Experimental Section” section.

**Table 4:** Physical and chemical quality of hydroponic recycled water after 14 days of epuvalisation treatment with fresh water, 14 days with 1:1 mixture brine:fresh water and other 21 days with 100% brine, compared to treatment with only fresh water during the same period. Mean values ± standard deviations (SD) of three replicates.

	100% Brine a			Fresh Water a		
		
Parameter	Influent b	Effluent c	% removal	Influent	Effluent	% removal
pH	7.20 ± 0.2	7.1 ± 0.1		7.02 ± 0.1	7.50 ± 0.1	
EC (mS cm^−1^)	6.04 ± 0.2	2.51 ± 0.1	58.0	2.1 ± 0.1	1.05 ± 0.1	50.0
TDS (mg L^−1^)	3,000 ± 100	1,560 ± 50.0	48.0	1,050 ± 50.0	510 ± 15.0	51.0
COD (mg L^−1^)	180 ± 20.0	60 ± 20.0	67.0	70 ± 15.0	38 ± 10.0	46.0
BOD (mg L^−1^)	85 ± 10.0	57 ± 10.0	12.0	38 ± 10.0	12 ± 5.00	68.0
Cl^−^ (mg L^−1^)	2,463 ± 200	1,676 ± 200	32.0	460 ± 10.0	337 ± 10.0	27.0
NO_3_^−^ (mg L^−1^)	1,017 ± 50.0	921 ± 15.0	10.0	545 ±25.0	44 ± 25.0	92.0
PO_4_^3−^ (mg L^−1^)	3.59 ± 0.3	0.94 ± 0.2	74.0	2.59 ± 0.1	0.94 ± 0.1	64.0
NH_4_^+^ (mg L^−1^)	688 ± 10.0	16 ± 10.0	98.0	41 ± 10.0	6 ± 4.00	85.0
Na^+^ (mg L^−1^)	329 ± 10.0	55 ± 5.0	83.0	58 ± 5.0	35 ± 5.0	40.0
K^+^ (mg L^−1^)	111 ± 25.0	45 ± 5.0	60.0	98 ± 1.0	9 ± 5.0	91.0
Ca^2+^ (mg L^−1^)	184 ± 10.0	163 ± 5.0	11.0	83 ± 2.0	78 ± 5.0	6.0
Mg^2+^ (mg L^−1^)	62 ± 5.0	46 ± 2.0	26.0	33 ± 5.0	31 ± 2.0	6.0

a Brine flowing from RO unit. Macro- and micro-nutrients were added as described in “Experimental Section” section;

b Composition of influent used as hydroponic water in the last 28 days;

c Composition of the effluent at the end of the whole experimental period (3 cycles).

## References

[1] Oron G., Gillerman L., Bick A., Mnaor Y., Buriakovsky N., Hagin J. (2007). Advanced low quality waters treatment for unrestricted use purposes:imminent challenges. Desalination.

[2] Al-Sajwani T.M.A., Lawrence R.J.

[3] Oron G., Gillerman L., Bick A., Buriakovsky N., Mnaor Y., Yitshak E.B., Katz L., Hagin J. (2006). A two stage membrane treatment of secondary effluent for unrestricted reuse and sustainable agriculture production. Desalination.

[4] Gillermana L., Bick A., Buriakovskya N., Oron G. (2006). Secondary wastewater polishing with ultrafiltration membranes for unrestricted reuse: Fouling and flushing modeling. Environ. Sci. Technol..

[5] Trivedy R.K. (2007). Low cost and energy saving technologies for water and wastewater treatment. J. Ind. Pollut. Control.

[6] Zhou H., Smith D.W. (2002). Advanced technologies in water and wastewater treatment. J. Environ. Eng. Sci.

[7] Goto T. (2002). East and South East Asia. Inst. Cheme.

[8] Oron G., Gillermana L., Buriakovskya N., Bickd A., Gargirb M., Dolanb Y., Manore Y., Katz L., Hagin J. (2008). Membrane technology for advanced wastewater reclamation for sustainable agriculture production. Desalination.

[9] Ng H.Y., Lee L.Y., Ong S.L., Tao G., Viawanath B., Kekre K., Lay W., Seah H. (2008). Treatment of RO brine-towards sustainable water reclamation practice. Water Sci. Technol.

[10] Arnal J.M., Sancho M., Iborra I., Gozalvez J.M., Santafe A., Lora J. (2005). Concentration of brines from RO desalination plants by natural evaporation. Desalination.

[11] Mushtaque A., Shayya W.H., Hoey D., Al-Handaly J. (2001). Brine Disposal from reverse osmosis desalination plants in Oman and United Arab Emirates. Desalination.

[12] Glater J., Cohen Y. Brine disposal from land based membrane desalination plants: A critical assessment.

[13] Smith D.J., Humphreys L. (2001). CSIRO Land and Water Sustainable Irrigated Agriculture Griffith 2000 Research Report.

[14] Mushtaque A., Arakel A., Hoey D., Thumarukudy M.R., Goosen M., Al-Haddabi M., Al-Belushi A. (2003). Feasibility of salt production from inland RO desalination plant reject brine: A case study. Desalination.

[15] Reimold R.J., Loland-McLaughlin G., Bloetscher F. (1996). An innovative opportunity for water reuse. Florida Water Resour. J.

[16] Mickley M. Environmental Considerations for the Disposal of Desalination Concentrate.

[17] Muchuweti M., Birkett J.W., Chinyanga E., Zvauya R., Scrimshaw M.D., Lester J.N. (2006). Heavy metal content of vegetables irrigated with mixture of wastewater and sewage sludge in Zimbabwe: Implications for human health. Agric. Ecosyst. Environ.

[18] Bahemuka T.E., Mubofu E.B. (1991). Heavy metals in edible green vegetables grown along the sites of the Sinza and Msimbazi rivers in Dar es Salaam, Tanzania. Food Chem.

[19] Mapanda F., Mangwayana E.N., Nyamangara J., Giller K.E. (2005). The effects of long-term irrigation using wastewater on heavy metal contents of soils under vegetables in Harare, Zimbabwe. Agric. Ecosyst. Environ.

[20] Everest W., Murphree T. (1995). Desalting residuals: A problem or a beneficial resource?. Desalination.

[21] Ahmed M., Shayya W.H., Hoey D., Mahendran A., Morris R., Al-Handaly J. (2000). Use of evaporation ponds for brine disposal in desalination plants. Desalination.

[22] Papadopoulos I., Chimonidou D., Savvides S., Polycarpou P. Optimization of Irrigation with Treated Wastewater on Flower Cultivations.

[23] Xanthoulis D., Dumont P., Wauthelet M. Epuvalisation: A Developing Technique. Experiences, results in different countries.

[24] Simon J., Bruce J. (2003). Membranes for Industrial Wastewater Recovery and Re-Use.

[25] Kowalski J.A., Palada M.C. (1995). Responce of selected vegetable crops to saline water in U.S Virgin Island.

[26] Ghaly A.E., Kamal M., Mahmoud N.S. (2005). Phytoremediation of aquaculture wastewater for water recycling and production of fish feed. Environ. Int.

[27] Mathieu J.J., Wang J.K., Timmons M.B. (1995). The Effect of Water Velocity and Nutrient Concentration on Plant Nutrient Uptake: A Review. Aquacultural Engineering and Waste Management. Proceedings from the Aquaculture Proceedings from the Aquaculture Expo VIII and Aquaculture in the Mid-Atlantic Conference.

[28] Rackocy J.E., Timmons M.B. (1995). The Role of Plant Crop Production in Aquaculture Waste Management. Aquaculutral Engineering and Waste Management: Proceedings from the Aquaculture Expo VIII and Aquaculture in the Mid-Atlantic Conference.

[29] Rackocy J.E., Hargreaves J.A., Wang J.K. (1993). Integration of Vegetable Hydroponics with Fish Culture: A Review. Techniques for Modern Aquaculture. Proceeding of an Aquacultural Engineering Conference.

[30] Jiang Z., Xinyuan Z. (1998). Treatment and utilization of wastewater in the Beijing zoo by an aquatic macrophyte system. Ecol. Eng.

[31] Batoul M.A., Nouf A.S. (2012). Evaluation of essential elements of sweet Basil (*Ocimum. Basilicum*) at different growth stages under deficit irrigation. Int. J. Appl. Biol. Pharm. Technol.

[32] Naegel L.C.A. (1997). Combined production of fish and plants in recirculating water. Aquaculture.

[33] Mant C., Peterkin J., May E., Butler J. (2003). A feasibility study of a *Salix viminalis* gravel hydroponic system to renovate primary settled wastewater. Bioresour. Technol.

[34] Marschner M. (1995). Mineral Nutrition of High Plants.

[35] Holtan H., Kamp-Nielsen L., Shuanes A.O. (1988). Phosphorous in soil, water and sediment: An overview. Hydrobiologia.

[36] Abbadi J., Gerendás J. (2012). Phosphorous use efficiency of Safflower and Sunflower studied in nutrient solutions. J. Agric. Sci. Technol.

[37] Brown J.J., Glenn E.P., Fitzsimmons K.M., Smith S.E. (1999). Halophytes for the treatment of saline aquaculture effluent. Aquaculture.

[38] Gloger K.C., Rakocy J.E., Conter J.B., Bailey D.S., Cole W.M., Shultz K.A., Timmons M.B. (1995). Contribution of lettuce to wastewater treatment capacity of raft hydroponics in a closed recirculating fish culture system. Aquacultural Engineering and Waste Management. Proceedings from the Aquaculture Expo VIII and Aquaculture in the Mid-Atlantic Conference.

[39] Dontje J.H., Clanton C.J. (1999). Nutrient fate in aquacultural systems for waste treatment. Trans. ASAE.

[40] Khalid K.A. (2006). Influence of water stress on growth, essential oil and chemical composition of herb (*Ocimum. basilicum* L.). Int. Agrophys.

[41] Westgate M.E., Grant L.T. (1989). Water deficits and reproduction in maize. Plant. Physiol.

[42] Khamis M., Karaman R., Ayyash F., Qtait A., Deeb O., Manassra A. (2011). Efficiency of advanced membrane wastewater treatment plant towards removal of aspirin, salicylic acid, paracetamol and p-aminophenol. J. Environ. Sci. Eng.

[43] Karaman R., Khamis M., Qurie M., Halabieh R., Makharzeh I., Mannassra A., Abbadi J., Qtait A., Bufo S.A., Nasser A. (2012). Removal of diclofenac potassium from wastewater using clay-micelle complex. Environ. Technol.

[44] Shekoofeh E., Sepideh H., Roya R. (2012). Role of mycorrhizal fungi and salicylic acid in salinity tolerance of Ocimum basilicum resistance to salinity. Afr. J. Biotechnol.

[45] Marotti M., Piccaglia R., Giovanelli E. (1996). Differences in essential oil composition of Basil (*Ocimum. basilicum* L.) Italian cultivars related to morfological characteristics. J. Agric. Food Chem.

[46] American Public Health Association (APHA) (2005). Standard Methods for Examination of Water and Wastewater Analysis.

[47] Ryan J., Harmsen S.G., Rashid A. (1996). A Soil and Plant Analysis Manual Adapted for the West Asia and North Africa Region.

[48] Gericke S., Kurmies B. (1952). Die kalorimetrische phosphorsäurebestimmung mit ammonium-vanadat-molybdat und ihre anwendung bei der pflanzenanalyse. Z pflanzenern düng Bodenkd.

[49] Searle P.L. (1984). The Berthelot or indophenol reaction and its use in the analytical chemistry of Nitrogen. Analyst.

